# ASO Author Reflections: Extended Pharmacologic Venous Thromboembolism Prophylaxis After Cancer Surgery—Provider Attitudes and Future Directions

**DOI:** 10.1245/s10434-025-18054-6

**Published:** 2025-08-20

**Authors:** Megan K. Scharner, Thomas Curran

**Affiliations:** 1 Medical University of South Carolina College of Medicine, Charleston, SC USA; 2 Division of Colon and Rectal Surgery, Department of Surgery, Medical University of South Carolina, Charleston, SC USA

## Past

Venous thromboembolism (VTE) is a rare but significant source of morbidity and mortality after cancer surgery. Extended pharmacologic VTE prophylaxis (ePPX) following major cancer surgery has been shown to be safe and effective and is recommended for up to 4 weeks postoperatively by major professional societies.^1^ Yet, national registry studies show that ePPX utilization is limited; approximately 5% following resection for colorectal and pancreatic cancer.^2,3^ While surveys of specialist surgeons report high guideline awareness and ePPX utilization,^4^ community surgeons have been poorly represented in the existing survey literature despite performing the majority of cancer operations in the USA.

## Present

We conducted semi-structured interviews with academic and community surgeons across a regional health network.^5^ Interviews characterized provider VTE perceptions, current prevention strategies, and barriers and facilitators to ePPX. An electronic medical record (EMR)-based decision support tool to improve ePPX utilization was demonstrated, and providers rated the tool’s usability. The cohort was composed of 13 surgeons and 2 advanced-practice providers practicing in academic and community settings. Provider ePPX practice pattern was categorized as “routine” (60%), “selective” (33%), or “never.” Academic providers were more likely to utilize ePPX routinely as compared with community providers. The most cited facilitator to ePPX utilization were society-based guidelines and evidence, while the most cited barrier to ePPX use was perceived patient comfort and fear. All providers were open to utilization of the EMR decision support tool.

## Future

Our qualitative study is novel in that it reflects the attitudes of surgeons practicing in both academic and community settings. Importantly, this work occurs in the context of a research program aiming to optimize guideline concordant ePPX utilization across a range of practice settings within a regional health network. These interviews informed the development of an EMR-based clinical decision support tool that has been deployed as part of an active stepped wedge randomized controlled trial in our health system. With the lessons learned from these surgeon interviews and ongoing patient interviews, we hope to refine interventions to decrease the incidence of VTE after cancer surgery. In partnership with the Wake Forest National Cancer Institute Community Oncology Research Program Research Base, we will be continuing this work with surveys and interviews of surgeons practicing in the community setting on a national scale. We hope this work will be foundational in improving outcomes for patients undergoing cancer surgery.
